# Validation of the German version of the insomnia severity index in adolescents, young adults and adult workers: results from three cross-sectional studies

**DOI:** 10.1186/s12888-016-0876-8

**Published:** 2016-05-31

**Authors:** Markus Gerber, Christin Lang, Sakari Lemola, Flora Colledge, Nadeem Kalak, Edith Holsboer-Trachsler, Uwe Pühse, Serge Brand

**Affiliations:** Department of Sport, Exercise and Health, University of Basel, St. Jakobsturm, Birsstrasse 320B, 4052 Basel, Switzerland; Department of Psychology, University of Warwick, Coventry, UK; Center for Affective, Stress and Sleep Disorders, Psychiatric Clinics of the University of Basel, Basel, Switzerland

**Keywords:** German, Insomnia, Measurement invariance, Psychological functioning, Sleep, Validation

## Abstract

**Background:**

A variety of objective and subjective methods exist to assess insomnia. The Insomnia Severity Index (ISI) was developed to provide a brief self-report instrument useful to assess people’s perception of sleep complaints. The ISI was developed in English, and has been translated into several languages including German. Surprisingly, the psychometric properties of the German version have not been evaluated**,** although the ISI is often used with German-speaking populations.

**Methods:**

The psychometric properties of the ISI are tested in three independent samples: 1475 adolescents, 862 university students, and 533 police and emergency response service officers. In all three studies, participants provide information about insomnia (ISI), sleep quality (Pittsburgh Sleep Quality Index), and psychological functioning (diverse instruments). Descriptive statistics, gender differences, homogeneity and internal consistency, convergent validity, and factorial validity (including measurement invariance across genders) are examined in each sample.

**Results:**

The findings show that the German version of the ISI has generally acceptable psychometric properties and sufficient concurrent validity. Confirmatory factor analyses show that a 1-factor solution achieves good model fit. Furthermore, measurement invariance across gender is supported in all three samples.

**Conclusions:**

While the ISI has been widely used in German-speaking countries, this study is the first to provide empirical evidence that the German version of this instrument has good psychometric properties and satisfactory convergent and factorial validity across various age groups and both men and women. Thus, the German version of the ISI can be recommended as a brief screening measure in German-speaking populations.

**Electronic supplementary material:**

The online version of this article (doi:10.1186/s12888-016-0876-8) contains supplementary material, which is available to authorized users.

## Background

Poor sleep is being increasingly recognised as a widespread and persistent health complaint. Estimates of prevalence of at least one symptom of insomnia stand at between 20 and 30 % in adult populations [1], with women typically having an increased risk of insomnia [2, 3]. A close relationship exists between people’s sleep, their daily well-being [4], memory [5], and daytime performance [6]. Moreover, chronic sleep complaints negatively impact on physical and psychological functioning in children [7], adolescents [8], adults [4], and the elderly [9]. For instance, a wealth of studies shows that sleep complaints are closely associated with depressive symptoms, with correlations ranging between *r* = .50 and .60 [10, 11].

A variety of objective and subjective methods exist to assess insomnia. Polysomnography and actigraphy are objective methods. They are considered reliable and valid techniques to assess sleep duration and efficiency [12]. However, polysomnography is costly, typically takes place in artificial sleep environments, and is not capable of detecting insomnia against subjective diagnostic criteria [9]. Among the subjective methods, sleep diaries are the most frequently used form of assessment [12, 13]. Although this method is cost-effective and correlates reasonably well with objective methods, sleep diaries depend on the willingness of participants to provide daily reports immediately after awakening over longer periods of time. In contrast, self-report questionnaires can be used to collect data on sleep problems with minimal effort and cost, while providing information about subjectively perceived consequences linked with sleep problems [9], such as irritability or difficulty concentrating. Given that insomnia constitutes a significant health hazard, which is often undetected and therefore undertreated, such reliable and economic tools are essential in facilitating early recognition and treatment of sleep complaints [14].

Because insomnia is a subjective disorder, Morin [15] developed the Insomnia Severity Index (ISI) to provide a brief self-report instrument useful to assess people’s subjective perception of sleep complaints in various populations. Thanks to its brevity (7 items, max. 2 min for completion and 1 min for scoring), the ISI can be used as a screening measure in clinical practice, allows for the assessment of change following treatment, and is also useful for epidemiological research. ISI scores enable a clinical evaluation with regards to insomnia symptoms. The ISI includes both nighttime and daytime components of insomnia and measures (a) the subjective symptoms and consequences of insomnia and (b) the concerns that result from these difficulties. In part, the ISI takes into account the diagnostic criteria of insomnia as formulated in the DSM-IV (Diagnostic and Statistical Manual of Mental Disorders, 4th Edition) by the American Psychiatric Association [16]. The instrument consists of seven items concerning the severity of sleep-onset (item 1) and sleep maintenance difficulties (including nocturnal and early morning awakening, items 2 and 3 respectively), current sleep satisfaction (item 4), interference of sleep difficulties with daily functioning (item 5), apparentness of impairment due to sleep complaints (item 6), and concerns or distress attributable to sleep complaints (item 7). All items are answered on a 5-point Likert scale ranging from 0 (not at all) to 4 (extremely), with a reference period of the previous 2 weeks. The ISI exists in three different versions: self-administered (patient), administered by a significant other (e.g., spouse, parent), and administered by a clinician [17, 18]. ISI scores range from 0 to 28 and can be interpreted as follows: 0–7 = no clinically significant insomnia, 8–14 = sub-threshold insomnia, 15–21 = clinical insomnia (moderate severity), and 22–28 = clinical insomnia (severe) [17, 19]. The suitability of these cut-offs has been tested both with classical test theory [17, 20] and item response theory [19]. The ISI has been used in clinical research and practice for almost 30 years.

The first systematic validation study was published in 2001 by Bastien et al. [17] showing that in a population of 145 patients suffering from clinical sleep disorders, the ISI had good internal consistency and concurrent validity with sleep diaries. As a result of treatment, the changes over time in the ISI were positively correlated with changes in sleep diaries, polysomnography and changes observed by the clinician.

Currently, several studies provide systematic support for the psychometric properties and the validity of the ISI in different populations such as insomnia patients [17, 19], young adults meeting the DSM-IV criteria for primary insomnia versus normal sleepers [20], community samples of adults [19, 21, 22], non-clinical child and adolescent populations [22, 23], elder care community day centre visitors [9], and cancer patients [18].

Regarding psychometric properties, all existing studies show that the ISI has acceptable internal consistency [9, 17, 19, 21–23], with Cronbach’s alphas generally exceeding the critical value of .70 [24]. Item-total correlations vary between .32 and .85 [9, 17, 19, 21, 22], with average item-total correlations above .50 [17, 19, 21]. Finally, one study showed that across a 2-week period the test-retest reliability of the ISI total score was satisfactory with a correlation of .79 [23].

With regards to external construct validity, prior research showed that ISI individual items correlated reasonably well with the corresponding variables on sleep diaries for sleep onset latency, waking after sleep onset and early morning awakening, with correlations between .11 and .91 [17, 19, 23]. As expected, significant, albeit weaker associations were found between the individual ISI variables and polysomnographic variables, with correlations ranging from .07 to .45 [17, 19]. Furthermore, moderate-to-strong relationships exist between the ISI and other self-report sleep questionnaires [9, 20, 23]. For instance, in Spanish adolescents, the correlation of the ISI total score with the Pittsburgh Sleep Quality Index (PSQI) was *r* = .68 [22]. Moreover, a good degree of convergence was found between the patient and clinician versions of the ISI, with correlations between *r* = .50 and .73 [17, 21, 23]. Additionally, significant correlations were observed between the ISI and other psychological constructs such as depressive symptoms [19, 22], anxiety [19, 22], general fatigue [19, 22], and psychological well-being [18, 19, 23].

The factorial validity of the ISI has been examined in several studies, mostly using exploratory factor analysis. These studies have provided inconsistent findings with solutions suggesting 1 to 3 factors [17–19]. So far, only the Spanish version of the ISI has been validated with confirmatory factor analysis (CFA) [22]. Fernandez-Mendoza et al. [22] compared three alternative models: Model 1 posited that all items load on a single factor (default model) [9, 25]. Model 2 assumed a two-factor structure with two correlated factors (factor 1 = night-time sleep difficulties: factor 2 = daytime impact of insomnia) [9, 18, 26]. Model 3 postulated a three-factor structure with three correlated factors (factor 1 = night-time sleep difficulties; factor 2 = sleep dissatisfaction; factor 3 = impact of insomnia). According to their data, Model 3 achieved the best model fit.

The ISI was developed in English [17, 19]. Subsequently, the ISI has been translated into several languages including French [18, 21], Spanish [22, 25], Chinese [9, 26], Korean [27], Persian [28], Hindi [29], and German [30, 31]. Surprisingly, to date, the psychometric properties of the German version have not been evaluated.

Given this background, the main objective of the present article was to validate the German version of the ISI across three age groups with a specific focus on gender invariance. This study is warranted for at least four reasons: First, the ISI has been widely used in sleep research during the last 15 years, including many studies with German-speaking samples [30, 32–35]. Second, German is one of the most frequently spoken languages worldwide with approximately 90 to 95 million first and 10 to 25 million second language speakers [36]. Third, although gender differences regarding the prevalence of insomnia symptoms are consistently reported in the literature [2, 3, 37], none of the previous studies has examined whether the psychometric properties of the ISI apply equally for women and men. Fourth, few validation studies have focused on younger people [22], and none of them has compared the validity of the ISI across different age groups. Nevertheless, such a comparison seems crucial to assure that an instrument is suitable for both younger and older populations.

Based on previous research, four hypotheses were formulated:

- Our first hypothesis was that female participants would report higher insomnia scores than their male counterparts [2, 3, 37].
- Our second hypothesis was that the ISI would have adequate homogeneity and internal consistency across all study populations, and both males and females [9, 17, 19, 21–23]. More specifically, we expected inter-item correlations ≥ .20, Cronbach’s alpha coefficients ≥ .70, and item-total correlations ≥ .30 [38].
- Our third hypothesis was that adequate convergent validity would exist in male and female participants across all study populations. That is, we hypothesized that the individual items and the total ISI score would be at least moderately and positively correlated with the corresponding items of the PSQI [17, 19, 23].
- Our fourth and last hypothesis was that the ISI total score would be at least moderately and positively correlated with indicators of psychological functioning [18, 19, 22, 23].
- With regard to factorial validity, we did not have a clear-cut hypothesis. Nevertheless, we expected that adequate model fit would be found with either a 1-, 2- or 3-factor model [17, 22]. For the best fitting model, we assumed fair factor loadings on the corresponding factors (≥ .45) [39], and at least weak measurement invariance across genders (see methods of study 1 for more information regarding types of measurement invariance).

## Study 1

### Methods

#### Participants and procedures

The sample consisted of 1475 adolescents (*M*_*age*_ = 13.4 years, *SD* = 1.4; range: 11–16 years; 49 % males) who were recruited from five middle schools in the German-speaking, north-western part of Switzerland. Data with this sample have been published previously [40]. Written informed consent was obtained from participants and parents, and the local ethics committee approved the study.

#### Sleep

To assess insomnia, the participants filled in the 7-item Insomnia Severity Index (ISI) [17], which has been described in detail in the introduction section. To ensure optimal translation, we rigorously followed the procedure set out by Brislin [41]. English items were translated into German, and then back-translated into English by an independent translator (see Additional file 1 for the wording of the items in German). To assess sleep quality, participants filled in a German adaptation [42] of the Pittsburgh Sleep Quality Index (PSQI) [43]. The PSQI includes several indicators to assess both sleep quality and sleep disturbances. The psychometric properties of the instrument are convincing [43]. The German version consists of 11 items, which concern two typical weekdays. The participants answered questions anchored on an 8-point Likert scale concerning sleep-related factors just after waking up in the morning (three items: perceived quality of sleep, restoration, and mood), during the daytime (two items: sleepiness and concentration), and before going to bed (two items: sleepiness and mood). Possible answers ranged from 1 (e.g., very bad sleep quality) to 8 (e.g., very good sleep quality). In addition, sleep onset latency (min), sleep duration and the number of awakenings during nighttime were assessed. Detailed information about bedtime and waking up allowed for the calculation of total sleep duration.

#### Psychological functioning

Psychological functioning was assessed with the KIDSCREEN-52 [44]. The questionnaire consists of 52 items focusing on 10 different domains of children’s and adolescents’ psychological functioning (e.g., psychological well-being, parent relation, etc.). Answers were given on a 5-point Likert scale, with the anchor points 1 (not at all) and 5 (extremely/always). The various domains can be aggregated to a global quality of life index, with higher mean scores reflecting higher psychological functioning (Cronbach’s alpha for the overall index = .92).

#### Statistical analyses

Univariate analyses of variance (ANOVA) were run to test gender differences. Product–moment correlations were calculated to examine homogeneity and item-total correlations. Cronbach’s alpha coefficients were obtained to test internal consistency. Correlational analyses were used to test convergent validity. Finally, CFA was used to test factorial validity. Although several models were tested in prior research [17, 22], we assumed that all items would load on the same factor. Thus, the 1-factor CFA model was based on seven observed measures and one latent construct. A default model was used, in which all parameters were freely estimated. This default model was then tested against a model, in which all free factor loadings were set equally across both genders. Parameter estimation was conducted using maximum likelihood (ML), and multiple goodness-of-fit indexes were considered to examine how well the theoretical model fitted the empirical data [45]. Measurement invariance across gender of the measurement model was tested via simultaneous multiple group comparison. Normed fit index (NFI) should be ≥ .95, probability of close fit (PCLOSE) ≥ .50, comparative fit index (CFI) ≥ .95, Tucker Lewis Index (TLI) ≥ .95, and root mean square error of approximation (RMSEA) ≤ .05. We first examined the most parsimonious 1-factor model. In case of unsatisfactory model fit, we continued with the more complex 2- and 3-factor models. According to Comrey and Lee [39], standardized factor loadings of ≥ .71 should be interpreted as excellent, ≥ .63 as very good, ≥ .55 as good, ≥ .45 as fair, and > .32 as poor. CFA are performed with AMOS® 22 (IBM Corporation, Armonk NY, USA), all other analyses with SPSS® 22 (IBM Corporation, Armonk NY, USA). To test measurement invariance across gender, we compared the default model against a model which assumed configural (same pattern of fixed and free factor loadings across groups), weak (invariant factor loadings across groups), strong (invariant factor loadings and intercepts across groups) and strict measurement invariance (invariant factor loadings, intercepts, and unique factor variances across groups) [46].

### Results

As shown in Table 1, the ISI mean score was 6.67 (*SD* = 4.39). In the total sample, 65 % (*n* = 963) of the participants reported no insomnia, 29 % (*n* = 431) sub-threshold insomnia, 4.7 % (*n* = 69) clinical insomnia of moderate severity, and 1 % (*n* = 12) severe clinical insomnia. Table 1 points out that girls (*M* = 7.23, *SD* = 4.46) reported more severe insomnia symptoms than boys (*M* = 6.08, *SD* = 4.24). Similarly, the Chi^2^-test showed that girls were overrepresented in the group with sub-threshold insomnia (32 % vs. 26 %) and moderate clinical insomnia (6 % vs. 3 %), whereas they were underrepresented in the group classified as having no insomnia (61 % vs. 70 %).

**Table 1:** Descriptive statistics of the total sample, test of gender differences, bivariate correlations between individual ISI items, and item-total correlations among male and female adolescents

	M	SD	Range	Skew	Kurt	Gender differences			
						ANOVA		Chi^2^-test	
Descriptive statistics						F	η^2^	Chi^2^	ϕ
Item 1	1.07	1.02	0-4	0.77	0.05	20.46***	.014	---	---
Item 2	0.65	0.97	0-4	1.51	1.60	7.40**	.005	---	---
Item 3	0.92	1.16	0-4	1.12	0.27	6.74*	.005	---	---
Item 4	1.03	0.96	0-4	0.94	0.50	25.80***	.017	---	---
Item 5	1.25	0.88	0-4	0.56	0.19	6.96**	.005	---	---
Item 6	1.14	0.97	0-4	0.84	0.46	3.44	.002	---	---
Item 7	0.61	0.89	0-4	1.61	2.43	11.40**	.008	---	---
ISI total score	6.67	4.39	0-27	1.00	1.13	25.91***	.017	21.02***	.12
Bivariate correlations	Item 1	Item 2	Item 3	Item 4	Item 5	Item 6	Item 7	r_it_	
Item 1	---	.43***	.27***	.58***	.25***	.17***	.39***	.55***	
Item 2	.39**	---	.47***	.53***	.17***	.11**	.34***	.55***	
Item 3	.25***	.43***	---	.36***	.13***	.12**	.28***	.42***	
Item 4	.53***	.51***	.36***	---	.25***	.14***	.50***	.63***	
Item 5	.23***	.24***	.16***	.34***	---	.47***	.30***	.39***	
Item 6	.15***	.14***	.11**	.20***	.46***	---	.17***	.28***	
Item 7	.42***	.34***	.25***	.48***	.33***	.27***	---	.52***	
r_it_	.50***	.54***	.39***	.64***	.44***	.32***	.54***	---	

Correlations for male participants are listed below the diagonal, correlations for girls above the diagonal. Item 1 = Difficulty falling asleep; Item 2 = Difficulty staying asleep; Item 3 = Waking up too early; Item 4 = How satisfied are you with your current sleep pattern?; Item 5 = To what extent do you consider your sleep problem to interfere with your daily functioning (e.g., daytime fatigue, ability to function at work/daily chores, concentration, memory, mood, etc.)?; Item 6 = How noticeable to others do you think your sleeping problem is in terms of impairing the quality of your life?; Item 7 = How worried/distressed are you about your current sleep problem?

*p < .05, **p < .01, ***p < .001

The majority of inter-item correlations exceeded the critical value of .20 in boys and girls (Table 1). Similarly, most item-total correlations were satisfactory, with an average correlation of .48. The lowest inter-item and item-total correlations were found for item 6 (*r*_*it*_ = .28 to .32). The Cronbach’s alpha was .76 for the total sample, boys and girls.

Regarding the associations between the ISI and PSQI items, significant correlations were found between item 1 and sleep onset latency, and item 2 and number of awakenings (Table 2). Contrary to our expectation, item 3 was not associated with sleep duration. Furthermore, a strong correlation was found between item 4 and sleep quality. Finally, significant correlations were found for items 5–7 with feeling restored and mood after awakening in the morning, as well as daytime sleepiness and concentration. The correlations between the ISI total score and the PSQI items show similar associations between the two instruments for boys and girls (Table 2). The moderate (negative) correlation between the ISI and psychological functioning provides further support for convergent validity of the ISI.

**Table 2:** Bivariate correlations between the ISI, the PSQI and psychological functioning among male and female adolescents

Males	Item 1	Item 2	Item 3	Item 4	Item 5	Item 6	Item 7	Total
**Sleep**								
SOL (min)	.42***	.27***	.17***	.27***	.13**	.08*	.17***	.38***
Awakenings	.27***	.36***	.24***	.32***	.19***	.08*	.24***	.34***
Sleep duration (h)	-.12**	-.04	***.01***	-.13***	-.02	-.08*	-.10**	-.10**
MR: Sleep quality	.54***	.46***	.34***	.71***	.38***	.23***	.54***	.71***
MR: Restoration	.37***	.28***	.21***	.55***	.35***	.29***	.41***	.54***
MR: Mood	.26***	.24***	.15***	.38***	.30***	.27***	.22***	.40***
DR: Sleepiness	.33***	.28***	.15***	.37***	.35***	.26***	.31***	.45***
DR: Concentration	.34***	.33***	.34***	.48***	.36***	.26***	.38***	.55***
ER: Sleepiness	-.01	.07	-.03	.03	.16***	.09*	.07	.08*
ER: Mood	.23***	.21***	.21***	.23***	.12**	.09*	.17***	.28***
**Psychological functioning**								
KIDSCREEN	-.30***	-.29***	-.23***	-.40***	-.23*	-.11	-.32***	-.38***
Females	Item 1	Item 2	Item 3	Item 4	Item 5	Item 6	Item 7	Total
**Sleep**								
SOL (min)	.47***	.27***	.22***	.40***	.19***	.09*	.31***	.44***
Awakenings	.26***	.49***	.30***	.32***	.13***	.05	.28***	.41***
Sleep duration (h)	-.10*	-.11**	***.04***	-.18***	-.11**	-.13***	-.08*	-.17***
MR: Sleep quality	.60***	.58***	.41***	.74***	.26***	.22***	.49***	.74***
MR: Restoration	.47***	.38***	.22***	.58***	.37***	.27***	.40***	.60***
MR: Mood	.32***	.24***	.13**	.31***	.32***	.33***	.22***	.41***
DR: Sleepiness	.37***	.36***	.23***	.39***	.43***	.32***	.35***	.54***
DR: Concentration	.37***	.35***	.28***	.44***	.33***	.23***	.37***	.53***
ER: Sleepiness	-.12**	.01	.00	.00	.12**	.15***	.05	***.04***
ER: Mood	.27***	.24***	.21***	.28***	.13***	.09**	.21***	.33***
**Psychological functioning**								
KIDSCREEN	-.37***	-.36***	-.26***	-.44***	-.28***	-.25***	-.34***	-.51***

Bold and bold italic coefficients are discussed in the text. Item 1 = Difficulty falling asleep; Item 2 = Difficulty staying asleep; Item 3 = Waking up too early; Item 4 = How satisfied are you with your current sleep pattern?; Item 5 = To what extent do you consider your sleep problem to interfere with your daily functioning (e.g., daytime fatigue, ability to function at work/daily chores, concentration, memory, mood, etc.)?; Item 6 = How noticeable to others do you think your sleeping problem is in terms of impairing the quality of your life?; Item 7 = How worried/distressed are you about your current sleep problem? *MR* morning rating, *DR* daytime rating, *ER* evening rating

*p < .05, **p < .01, ***p < .001

**Fig. 1:**
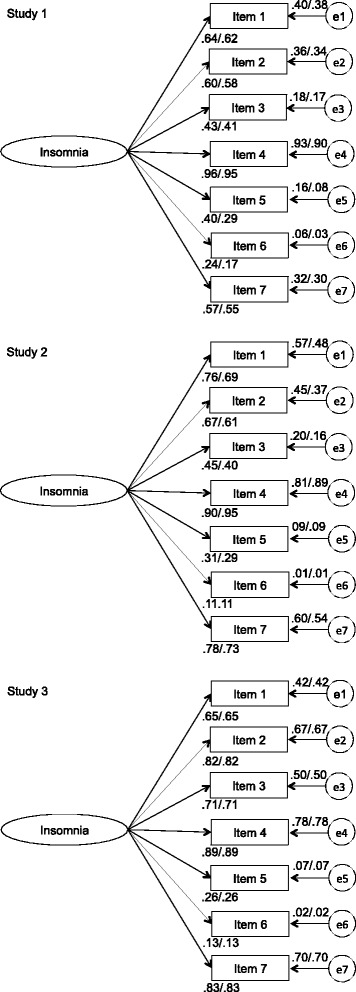
Factor Loadings For Confirmatory Factor Analysis for Males (First Coefficient) and Females (Second Coefficient), Separately for Study 1, 2 and 3

With regard to factorial validity, as shown in Table 3, the model fit of the initial 1-factor model was excellent. Furthermore, Table 3 provides support for configural and weak measurement invariance (invariant factor loadings) across genders, *p*(Δχ^2^) = .28. Most of the factor loadings were fair to excellent, except for items 5 and 6 with loadings ranging from .17 to .40. Figure 1 provides the measurement coefficients of the hypothesized 1-factor model (after testing for configural and weak invariance), separately for boys and girls.

**Table 3:** Goodness-of-fit indices and model comparison

Study 1	CFI	TLI	NFI	PClose	RMSEA	*p*(Δχ^2^)
1-Factor Model^a^						
Default model	0.98	0.96	0.98	0.94	.04 (.03, .05)	---
+ Configural invariance across genders	0.98	0.96	0.97	0.96	.04 (.03, .05)	.084
+ Weak invariance across genders	0.98	0.97	0.97	0.99	.04 (.03, .05)	.284
+ Strong invariance across genders	---	---	---	---	---	.000
+ Strict invariance across genders	---	---	---	---	---	---
Study 2	CFI	TLI	NFI	PClose	RMSEA	*p*(Δχ^2^)
1-Factor Model^1^						
Default model	1.00	1.00	1.00	1.00	.00 (.00, .03)	---
+ Configural invariance across genders	1.00	1.00	1.00	1.00	.01 (.00, .03)	.072
+ Weak invariance across genders	1.00	1.00	1.00	1.00	.02 (.00, .04)	.058
+ Strong invariance across genders	1.00	1.00	1.00	1.00	.02 (.00, .04)	.124
+ Strict invariance across genders	---	---	---	---	---	.003
Study 3	CFI	TLI	NFI	PClose	RMSEA	*p*(Δχ^2^)
1-Factor Model^a^						
Default model	1.00	1.00	0.99	0.99	.00 (.00, .04)	---
+ Configural invariance across genders	1.00	1.00	0.99	0.98	.00 (.00, .04)	.121
+ Weak invariance across genders	1.00	1.00	0.98	0.98	.00 (.00, .04)	.116
+ Strong invariance across genders	1.00	1.00	0.98	1.00	.00 (.00, .04)	.855
+ Strict invariance across genders	1.00	1.00	0.97	1.00	.00 (.00, .04)	.417

*AGFI* adjusted goodness of fit index, *CFI* comparative fit index, *TLI* Tucker Lewis index, *RMR* root mean square residual, *PClose* probability of close fit, *RMSEA* root mean square error of approximation

^a^e1-e2, e1-e3, e2-e3, e5-e6, e5-e7, e6-7 were allowed to correlate

## Study 2

### Methods

#### Participants and procedures

Sample 2 consisted of 862 students (*M*_age_ = 24.7, *SD* = 5.9) from the German-speaking, north-western part of Switzerland (223 men, 639 women), who were recruited from the University of Basel (*n* = 556) and from the Northwestern University of Applied Sciences (*n* = 306). Data with this sample have been published previously [31, 47, 48]. Participants provided informed consent and the local ethics committee approved the study.

#### Sleep

As in study 1, sleep complaints were assessed with the ISI, quality of sleep with the 11-item German adaptation of the PSQI.

#### Psychological functioning

Psychological functioning was assessed via depressive symptoms, using the Depression Scale (DS) [49]. This scale consists of 16 items ranging from 1 (not at all true) to 4 (definitely true) and concerns decreased mood, lack of satisfying social and leisure activities, thoughts about suicide, and hopelessness. The internal consistency of the DS proved to be good in the present sample (Cronbach’s alpha = .86).

#### Statistical analyses

The same statistical procedures as those from study 1 were used.

### Results

The ISI mean score was 6.56 (*SD* = 4.31) in the total sample. Moreover, 68 % (*n* = 585) were categorized as having no insomnia, 26 % (*n* = 224) as having sub-threshold insomnia, 6 % (*n* = 49) as having moderate clinical insomnia, and 1 % (*n* = 4) as having severe clinical insomnia. The ANOVA (Table 4) shows that women (*M* = 6.80, *SD* = 4.37) reported more insomnia symptoms than men (*M* = 5.91, *SD* = 4.13). Gender differences were found for items 1 and 2, but not for the other items. The Chi^2^-test did not detect differences with regard to insomnia categories between men and women.

**Table 4:** Descriptive statistics of the total sample, test of gender differences, bivariate correlations between individual ISI items, and item-total correlations among male and female young adults

	M	SD	Range	Skew	Kurt	Gender differences			
						ANOVA		Chi^2^-test	
Descriptive statistics						F	η^2^	Chi^2^	ϕ
Item 1	0.67	0.93	0-4	1.45	1.76	5.67*	.007	---	---
Item 2	0.66	0.92	0-4	1.47	1.80	9.46**	.011	---	---
Item 3	0.49	0.86	0-4	1.90	3.13	0.95	.001	---	---
Item 4	1.09	1.04	0-4	0.82	0.03	2.61	.003	---	---
Item 5	1.71	1.01	0-4	0.28	−0.57	1.63	.002	---	---
Item 6	1.38	1.01	0-4	0.34	−0.56	1.79	.002	---	---
Item 7	0.57	0.91	0-4	1.57	1.64	1.48	.002	---	---
ISI total score	6.56	4.31	0-26	1.09	1.35	6.88**	.008	4.67	.073
Bivariate correlations	Item 1	Item 2	Item 3	Item 4	Item 5	Item 6	Item 7	r_it_	
Item 1	---	.40***	.20**	.63***	.19**	.05	.58***	.53***	
Item 2	.39***	---	.42***	.59***	.16*	.11	.47***	.55***	
Item 3	.30***	.48***	---	.36***	.10	.10	.29***	.36***	
Item 4	.67***	.51***	.41***	---	.22**	.10	.70***	.68***	
Item 5	.28***	.20***	.17***	.26***	---	.52***	.29***	.37***	
Item 6	.06	.07	.06	.09*	.47***	---	.15*	.25***	
Item 7	.56***	.47***	.32***	.64***	.42***	.25***	---	.66***	
r_it_	.57***	.52***	.42***	.65***	.45***	.25***	.69***	---	

Correlations for male participants are listed below the diagonal, correlations for girls above the diagonal. Item 1 = Difficulty falling asleep; Item 2 = Difficulty staying asleep; Item 3 = Waking up too early; Item 4 = How satisfied are you with your current sleep pattern?; Item 5 = To what extent do you consider your sleep problem to interfere with your daily functioning (e.g., daytime fatigue, ability to function at work/daily chores, concentration, memory, mood, etc.)?; Item 6 = How noticeable to others do you think your sleeping problem is in terms of impairing the quality of your life?; Item 7 = How worried/distressed are you about your current sleep problem?

*p < .05, **p < .01, ***p < .001

As shown in Table 4, the inter-item correlations were mostly above the critical value of .20. Acceptable item-total correlations were also found for men and women (with mean correlations of .51 for men and .49 for women). The Cronbach’s alpha was .77 in the total sample, .78 for men and .76 for women.

With regard to the ISI-PSQI correlations, significant associations occurred for item 1 and sleep onset latency, and item 2 and number of awakenings (Table 5). As in study 1, no significant relationship was found to exist between item 3 and sleep duration. Furthermore, a strong correlation was identified between item 4 and sleep quality. Significant correlations also existed between items 5 and 7 and feeling restored in the morning, as well as daytime sleepiness and concentration. The correlations for item 6 pointed into the same direction, but were generally weak. The correlations between the PSQI items and the ISI revealed similar relationships for men and women. Finally, Table 5 revealed that insomnia is strongly associated with depressive symptoms, independent of participants’ gender.

**Table 5:** Bivariate Correlations Between the ISI, the PSQI and Psychological Functioning among Male and Female Young Adults

Males	Item 1	Item 2	Item 3	Item 4	Item 5	Item 6	Item 7	Total
**Sleep**								
SOL (min)	.51***	.11	.02	.42***	.07	.08	.32***	.34***
Awakenings	.34***	.57***	.35***	.41***	.15*	.04	.33***	.46***
Sleep duration (h)	.13	-.06	***-.04***	.10	.09	.05	.10	***.09***
MR: Sleep quality	.39***	.48***	.24***	.55***	.23**	.06	.48***	.54***
MR: Restoration	.41***	.26***	.23**	.54***	.24**	***.07***	.43***	.49***
MR: Mood	.13	.05	-.01	.18**	***.08***	***.01***	.18**	.14*
DR: Sleepiness	.30***	.26***	.25***	.46***	.44***	.21**	.41***	.52***
DR: Concentration	.36***	.29***	.29***	.47***	.43***	***.10***	.49***	.53***
ER: Sleepiness	-.33***	-.03	-.01	-.30***	-.02	.02	-.21	-.19***
ER: Mood	.12	.15*	.14*	.26***	.16*	.10	.24***	.26***
**Psychological functioning**								
Depression scale	.36***	.35***	.38***	.40***	.31***	.10	.43***	.50***
Females	Item 1	Item 2	Item 3	Item 4	Item 5	Item 6	Item 7	Total
**Sleep**								
SOL (min)	.53***	.25***	.13**	.43***	.15***	.10*	.40***	.44***
Awakenings	.19***	.47***	.25***	.38***	.08	.11**	.25***	.38***
Sleep duration (h)	.09***	-.08	***-.03***	.06	.00	-.03	-.02	***.00***
MR: Sleep quality	.51***	.51***	.33***	.69***	.24***	.12**	.57***	.65***
MR: Restoration	.46***	.38***	.22***	.54***	.25***	.14***	.53***	.56***
MR: Mood	.26***	.18***	-.04	34***	.20***	.16***	.32***	.34***
DR: Sleepiness	.35***	.31***	.21***	.41***	.27***	.13***	.45***	.47***
DR: Concentration	.37***	.34***	.27***	.40***	.29***	.16***	.45***	.51***
ER: Sleepiness	-.15***	.03	.02	-.09*	-.02	-.03	-.08	***-.07***
ER: Mood	.25***	.17***	.13**	.23***	.04	.02	.21***	.23***
**Psychological functioning**								
Depression scale	.35***	.35***	.27***	.38***	.27***	.19***	.46***	.51***

Bold and bold italic coefficients are discussed in the text. Item 1 = Difficulty falling asleep; Item 2 = Difficulty staying asleep; Item 3 = Waking up too early; Item 4 = How satisfied are you with your current sleep pattern?; Item 5 = To what extent do you consider your sleep problem to interfere with your daily functioning (e.g., daytime fatigue, ability to function at work/daily chores, concentration, memory, mood, etc.)?; Item 6 = How noticeable to others do you think your sleeping problem is in terms of impairing the quality of your life?; Item 7 = How worried/distressed are you about your current sleep problem? *MR* morning rating, *DR* daytime rating, *ER* evening rating

*p < .05, **p < .01, ***p < .001

The findings of the CFA confirm that the 1-factor model fits well with the empirical data (Table 3). Strong measurement invariance (invariant factor loadings and intercepts) across genders was supported. Most factor loadings were fair to excellent. However, the factor loadings of item 5 (.29 to .31) and 6 (.11) were poor (Fig. 1).

## Study 3

### Methods

#### Participants and procedures

Sample 3 consisted of 533 employees of the police force and emergency response service corps in the German-speaking, north-western part of Switzerland (*M*_*age*_ = 41.2, *SD* = 9.8 years, 411 men and 122 women), who responded to a written questionnaire (45 % return rate). Data with this sample have been published previously [50, 51]. Participants gave informed consent and the study was performed in accordance with the ethical standards laid down in the Declaration of Helsinki.

#### Sleep

The same instruments as in study 1 and 2 were used to measure sleep complaints (ISI) and quality of sleep (PSQI).

#### Psychological functioning

To assess psychological functioning, participants completed the 12-Item Short Form Health Survey (SF-12) [52]. The composite score for the psychological subscale was obtained by weighting each item as described in the SF-12 manual. Higher scores reflect increased health functioning.

#### Statistical analyses

The same statistical procedures were used as in study 1.

### Results

As shown in Table 6, the ISI mean score was 6.98 (*SD* = 4.96) in the total sample. Moreover, 62 % (*n* = 331) of the participants had no insomnia, 30 % (*n* = 160) had sub-threshold insomnia, 7 % (*n* = 35) had clinical insomnia of moderate severity, whereas 1 % (*n* = 7) had severe clinical insomnia. Unlike study 1 and 2, women (*M* = 7.00, SD = 5.20) and men (*M* = 6.97, *SD* = 4.89) did not differ with regard to any of the insomnia measures (Table 7).

**Table 6:** Descriptive Statistics of the Total Sample, Test of Gender Differences, Bivariate Correlations Between Individual ISI Items, and Item-Total Correlations Among Male and Female Adult Workers

	M	SD	Range	Skew	Kurt	Gender differences			
						ANOVA		Chi^2^-test	
Descriptive statistics						F	η^2^	Chi^2^	ϕ
Item 1	0.70	0.93	0-4	1.27	0.95	2.70	.005		
Item 2	1.09	1.15	0-4	0.93	0.09	0.05	.000	---	---
Item 3	0.99	1.11	0-4	0.94	−0.01	0.40	.001	---	---
Item 4	1.41	1.07	0-4	0.55	−0.40	0.09	.000	---	---
Item 5	1.23	0.95	0-4	0.58	−0.18	0.34	.001	---	---
Item 6	0.89	0.92	0-4	0.80	−0.17	0.38	.001	---	---
Item 7	0.67	1.01	0-4	1.52	1.56	0.00	.000	---	---
ISI total score	6.98	4.96	0-26	1.02	0.92	0.01	.000	0.48	.030
Bivariate correlations	Item 1	Item 2	Item 3	Item 4	Item 5	Item 6	Item 7	r_it_	
Item 1	---	.40***	.32***	.51***	.20***	.12*	.49***	.49***	
Item 2	.56***	---	.72***	.71***	.23***	.13*	.66***	.72***	
Item 3	44***	.57***	---	.62***	.20***	.11*	.61***	.65***	
Item 4	.63***	.73***	.51***	---	.19***	.06	.70***	.70***	
Item 5	.20*	.15	.22*	.19*	---	.45***	.30***	.35***	
Item 6	.12	.06	.05	.12	.41***	---	.27***	.25***	
Item 7	.62***	.66***	.54***	.75***	.40***	.22*	---	.77***	
r_it_	.64***	.68***	.57***	.73***	.34***	.21*	.68***	---	

Correlations for male participants are listed below the diagonal, correlations for girls above the diagonal. Item 1 = Difficulty falling asleep; Item 2 = Difficulty staying asleep; Item 3 = Waking up too early; Item 4 = How satisfied are you with your current sleep pattern?; Item 5 = To what extent do you consider your sleep problem to interfere with your daily functioning (e.g., daytime fatigue, ability to function at work/daily chores, concentration, memory, mood, etc.)?; Item 6 = How noticeable to others do you think your sleeping problem is in terms of impairing the quality of your life?; Item 7 = How worried/distressed are you about your current sleep problem?

*p < .05, ***p < .001

**Table 7:** Bivariate Correlations Between the ISI, the PSQI and Psychological Functioning among Male and Female Adult Workers

Males	Item 1	Item 2	Item 3	Item 4	Item 5	Item 6	Item 7	Total
**Sleep**								
SOL (min)	.51***	.27***	.21***	.28***	.10*	.13**	.25***	.36***
Awakenings	-.18***	.52***	.46***	.45***	.18***	.05	.37***	.47***
Sleep duration (h)	-.20***	-.20***	-.20***	-.27***	-.04	-.02	-.23***	-.25***
MR: Sleep quality	.48***	.65***	.56***	.69***	.28***	.18***	.65***	.74***
MR: Restoration	.40***	.47***	.39***	.56***	.36***	.23***	.53***	.61***
MR: Mood	.31***	.30***	.22***	.37***	.33***	.21***	.35***	.42***
DR: Sleepiness	.32***	.36***	.34***	.45***	.40***	.27***	.45***	.53***
DR: Concentration	.37***	.42***	.44***	.43***	.39***	.22***	.45***	.56***
ER: Sleepiness	-.07	.12*	.17**	.06	.06	.08	.10*	.11*
ER: Mood	.26***	.25***	.25***	.29***	.17***	.07	.31***	.33***
**Psychological functioning**								
SF12	-.27***	-.35***	-.35***	-.37***	-.25***	-.20***	-.42***	-.46***
Females	Item 1	Item 2	Item 3	Item 4	Item 5	Item 6	Item 7	Total
**Sleep**								
SOL (min)	.28**	.22*	.21*	.34***	.06	.07	.32***	.31***
Awakenings	.34***	.63***	.36***	.55***	.16	.03	.48***	.54***
Sleep duration (h)	-.25**	-.27**	-.18*	-.10	.04	.04	-.11	-.18*
MR: Sleep quality	.50***	.63***	.47***	.74***	.35***	.13	.73***	.75***
MR: Restoration	.37***	.44***	.31***	.62***	.29***	.20*	.58***	.59***
MR: Mood	.29**	.23*	.27**	.38***	.24**	***.14***	.34***	.39***
DR: Sleepiness	.28**	.44***	.31***	.46***	.36***	***.11***	.53***	.52***
DR: Concentration	.13	.23*	.31*	.33***	.36***	***.15***	***.33***	.36***
ER: Sleepiness	.13	.01	.08	.01	.13	.12	.06	***.05***
ER: Mood	.23**	.25**	.30**	.34***	.17	.06	.33***	.33***
**Psychological functioning**								
SF12	-.22*	-.35*	-.30**	-.45***	-.27**	-.07	-.41***	-.43***

Bold and bold italic coefficients are discussed in the text. Item 1 = Difficulty falling asleep; Item 2 = Difficulty staying asleep; Item 3 = Waking up too early; Item 4 = How satisfied are you with your current sleep pattern?; Item 5 = To what extent do you consider your sleep problem to interfere with your daily functioning (e.g., daytime fatigue, ability to function at work/daily chores, concentration, memory, mood, etc.)?; Item 6 = How noticeable to others do you think your sleeping problem is in terms of impairing the quality of your life?; Item 7 = How worried/distressed are you about your current sleep problem? *MR* morning rating, *DR* daytime rating, *ER* evening rating

*p < .05, **p < .01, ***p < .001

The majority of inter-item correlations exceeded the critical value of .20 for most pairs of items, independent of participants’ gender. Similarly, most item-total correlations were satisfactory (with average correlations of .55 for men and .56 for women). The Cronbach’s alpha was .81 in the total sample, .81 for men and .82 for women.

With regard to the associations between the ISI and PSQI items, significant correlations existed between item 1 and sleep onset latency, item 2 and number of awakenings, and item 3 and sleep duration (Table 7). Furthermore, a strong correlation was found between item 4 and sleep quality. Significant correlations also existed between items 5 and 7 and feeling restored and mood after awakening in the morning, as well as daytime sleepiness and concentration. The correlations for item 6 pointed into the same direction, but were generally weak. Similar associations were found among men and women with regard to the correlations between the PSQI items and the ISI total score. The significant negative correlations between the ISI and the SF12 psychological functioning scale further support the convergent validity of the instrument (Table 7).

The CFA corroborated that a 1-factor model had excellent model fit. Furthermore, strict measurement invariance (invariant factor loadings, intercepts, and unique factor variances across groups) was supported across genders (Table 3). Five of seven factor loadings were very good or excellent. Nevertheless, the factor loadings of items 5 and 6 were poor (between .13 and .26; see Fig. 1).

## Discussion

The key findings of the present study are that the German version of the ISI has generally acceptable psychometric properties and sufficient concurrent validity to be recommended as a brief screening measure in both adolescents and adults. Moreover, the factor structure of the ISI proved to be invariant across gender.

Four hypotheses were formulated and each of these will now be discussed in turn.

Our first hypothesis was that female participants would report higher insomnia scores than their male counterparts. This hypothesis was supported in adolescents and young adults, which is consistent with the majority of previous studies [2, 3]. Contrary to our hypothesis, no gender differences were found in police and emergency service response officers. A meta-analysis of 29 studies showed that a female predisposition to insomnia is consistent and progressive across age [53]. Thus, increasing age does not provide a plausible explanation. Most likely, this unexpected finding can be attributed to the fact that in study 3 men were overrepresented in shift workers in this specific professional group [50], which might contribute to increased insomnia mean scores among male participants [54]. Further, it is also conceivable that the job of police officer per se led to a selection bias.

Our second hypothesis was that the ISI would have adequate homogeneity and internal consistency across all study populations, and both men and women [9, 17, 19, 21–23]. This hypothesis was generally supported. First, all Cronbach’s alpha coefficients were ≥ .70 for both men and women [38]. Second, across all three samples, the majority of inter-item correlations were ≥ .20, and most item-total correlations were ≥ .30. The lowest coefficients were consistently shown for item 6. This is congruent with previous research, and most likely due to the fact that this item refers to the opinion of others about one’s own sleep. Thus, information about what others think might not be as subjectively relevant as individual perceptions about one’s own sleep. Not surprisingly, therefore, item 6 also had somewhat suboptimal factor loadings in the CFA, both in male and female participants. Nevertheless, we decided not to exclude this item since the low factor loading did not negatively affect the general model fit, and because exclusion of item 6 would not have resulted in substantial improvements in the Cronbach’s alpha coefficient.

In our third hypothesis, we assumed that adequate convergent validity would exist for male and female participants throughout all study populations. This hypothesis was fully supported. In line with previous research, our findings show that the individual items and the total ISI score correlate at least moderately and positively with the corresponding PSQI items [17, 19, 23]. Moreover, our data corroborate previous research showing that the ISI is associated with impaired psychological functioning [18, 19, 22, 23].

With regard to factorial validity, the findings of our studies point out that a 1-factor model provides an excellent model fit across all age groups. We acknowledge that the 2- and 3-factor models also had very good model fit (data not shown). For several reasons, however, the 1-factor model seems the most suitable one: First, it is generally recommended to use the most parsimonious model if goodness-of-fit indices are satisfied by several alternative models [55]. Second, the 2- and 3-factor models showed very high inter-factor correlations with coefficients ranging between .60 and .98. This reveals a great overlap between the latent factors, which might pose problems associated with multicollinearity when using the separate factors as independent predictors. Third, the loadings of item 5 and 6 on the latent factor did not improve substantially in the multifactorial models. Fourth, in the 3-factor model, one factor consists of one item only (factor 2: satisfaction), which precludes a test of internal consistency. Furthermore, this single item factor proved to have substantial cross-loadings on the other two factors in previous research [22]. Fifth, cut-offs scores to establish the severity of insomnia only exist for the total score [19].

Finally, weak-to-strict measurement invariance across all age groups was supported. This is an important finding because Widaman et al. [46] emphasized that if participants’ answers vary so much with age that significant differences emerge in the factor structure of an instrument, or that relevant ceiling or floor effects occur at different ages, the measuring devices must change. The present analyses suggest that the ISI is equally suitable to detect insomnia symptoms in adolescents, young adults and adult workers.

The findings of the present study need to be interpreted in light of several limitations: First, the findings are based on cross-sectional data. Thus, we were unable to test predictive validity and the test-retest reliability. Nevertheless, a previous study with a German-speaking sample of high school students showed that the ISI score improved after 3 weeks of daily morning running [56]. Moreover, we were not able to test measurement invariance across time within the same individuals [46]. Second, the findings are entirely based on self-reported data. Thus, we did not test the degree to which the ISI items correlated with objective sleep measures. Nevertheless, the German version of the ISI proved to be weakly but significantly associated with 1-channel EEG measures in previous studies [34], with a magnitude of relationships similar to that reported in the international literature [17, 19]. Third, all three studies used non-clinical populations, which precludes the establishment of the discriminant validity of the ISI. Nevertheless, previous research showed that the ISI discriminates well between patients with insomnia and controls without sleep disorders [19–21, 23], and offers a good balance between sensitivity (likelihood of detecting insomnia in a subject from the insomnia group) and specificity (likelihood of rejecting insomnia in a subject from the control group) [20–22]. Fourth, we acknowledge that a clinical evaluation should be seen as the gold standard for the detection of sleep difficulties [19]. However, a clinical evaluation is time-consuming, which may discourage general practitioners from systematically inquiring about sleep in their patients [19]. Thus, the ISI provides a time- and cost-effective alternative, which seems feasible for general practitioners and for public health screening purposes.

## Conclusions and practical relevance

While the ISI has been widely used in German-speaking countries, this study is the first to provide empirical evidence that the German version of the instrument has good psychometric properties and satisfactory convergent and factorial validity across various age groups and both men and women. Compared to other instruments which identify and quantify sleep disturbances [43, 57], the ISI is brief and cheap, easy to administer and to score, and corresponds well with the diagnostic criteria for insomnia defined in the DSM-IV. Thus, the tool provides relevant information for diagnosis and treatment planning and allows assessment of the clinical significance of the complaints by providing cut-off scores.

## Abbreviations

ANOVA, analysis of variance; CFA, confirmatory factor analysis; CFI, comparative fit inex; DS, depression scale; DSM-IV, diagnostic and statistical manual of mental disorders; ISI, insomnia severity index; ML, maximum likelihood; NFI, normed fit index; PClose, probability of close fit; PSQI, pittsburgh sleep quality index (psqi); RMSEA, root mean square error of approximation; SF-12, 12-item short form health survey; TLI, Tucker Lewis Index

## Additional file

Additional file 1: Validation of the German Version of the Insomnia Severity Index_Supplementary Online Material. Insomnia Severity Index – Deutsche Übersetzung (German translation). This file provides the German translation of the Insomnia Severity Index, as well as a guide for (a) calculating an overall index and (b) interpreting the overall score. (DOCX 81 kb)
