# Supplementary material for: Validation of the German version of the insomnia severity index in adolescents, young adults and adult workers: results from three cross-sectional studies
Source: BMC Psychiatry. 2016 May 31;16:174. doi: 10.1186/s12888-016-0876-8 (PMC4888604; doi:10.1186/s12888-016-0876-8)
Supplement: Additional file 1: — Validation of the German Version of the Insomnia Severity Index_Supplementary Online Material. Insomnia Severity Index – Deutsche Übersetzung (German translation). This file provides the German translation of the Insomnia Severity Index, as well as a guide for (a) calculating an overall index and (b) interpreting the overall score. (DOCX 81 kb) [file 12888_2016_876_MOESM1_ESM.docx]

**Insomnia Severity Index – Deutsche Übersetzung (German translation)**

Bitte schätzen Sie ein, inwiefern Ihr Schlaf **in den letzten beiden Wochen** beeinträchtigt wurde durch ...

|  |  | gar nicht | leicht | mittel | schwer | sehr schwer |
| --- | --- | --- | --- | --- | --- | --- |
| 1. | Einschlafstörungen | (0) | (1) | (2) | (3) | (4) |
| 2. | Durchschlafstörungen | (0) | (1) | (2) | (3) | (4) |
| 3. | Früherwachen | (0) | (1) | (2) | (3) | (4) |

|  |  | sehr zufrieden | zu-frieden | neutral | unzu-frieden | sehr unzufrieden |
| --- | --- | --- | --- | --- | --- | --- |
| 4. | Wie zufrieden/unzufrieden sind Sie mit Ihrem gegenwärtigen Schlaf? | (0) | (1) | (2) | (3) | (4) |

|  |  | überhaupt nicht | ein  wenig | mässig | stark | sehr  stark |
| --- | --- | --- | --- | --- | --- | --- |
| 5. | Wie stark glauben Sie, dass andere Personen die Auswirkung Ihres (schlechten) Schlafes auf Ihre Lebensqualität wahrnehmen? | (0) | (1) | (2) | (3) | (4) |
| 6. | Inwiefern macht Ihnen Ihr (schlechter) Schlaf zur Zeit Sorgen? | (0) | (1) | (2) | (3) | (4) |
|  |  |  |  |  |  |  |
| 7. | Wie stark ist Ihre Leistungsfähigkeit (z.B. Konzentration, Gedächtnis) und Ihr Wohlbefinden (z.B. Müdigkeit, Stimmung) tagsüber durch Ihren (schlechten) Schlaf beeinträchtigt? | (0) | (1) | (2) | (3) | (4) |

**Anleitung zur Berechnung des Insomnia Severity Index**

Summe aus allen sieben Items bilden: 1+2+3+4+5+6+7 = ____________ (Gesamtwert)

**Interpretation des Gesamtwerts:**

0-7: Keine klinisch bedeutsame Insomnie

8-14: Unterschwellige klinische Insomnie

15-21: Klinisch bedeutsame Insomnie (moderate Ausprägung)

22-28: Klinisch bedeutsame Insomnie (starke Ausprägung)
